# Aortic Regurgitation: From Valvular to Myocardial Dysfunction

**DOI:** 10.3390/jcm13102929

**Published:** 2024-05-16

**Authors:** Alba-Nidia Marigliano, José-Tomas Ortiz, Jorge Casas, Arturo Evangelista

**Affiliations:** 1 Heart Institute, Teknon Medical Center, 08022 Barcelona, Spain; alba.nidia@quironsalud.es (A.-N.M.); jtortiz@clinic.cat (J.-T.O.); 2 Instituto Cedic, Bahía Blanca B8000, Argentina; jorgecasas31@hotmail.com

**Keywords:** aortic regurgitation, Doppler echocardiography, magnetic resonance imaging, left ventricular dysfunction, myocardial fibrosis, valvular surgical treatment

## Abstract

Chronic aortic regurgitation (AR) leads to volume overload in the left ventricle (LV), which is well tolerated for years. In this condition, the LV usually dilates with minimal reduction in the ejection fraction (EF), even in the absence of symptoms. Echocardiography is the primary imaging test used to quantify AR. However, no single assessment of Doppler measures is accurate and precise in individual patients; therefore, the integration of multiple parameters is necessary. Recent guidelines recommend surgical treatment for severe AR in patients who are symptomatic or have an LVEF < 55% and an end-systolic diameter > 50 mm. Nevertheless, advances in imaging technology have improved the quantification of AR and the assessment of LV subclinical dysfunction. It is widely recognized that patients who undergo aortic valve replacement/repair (AVR) due to symptoms or a low LVEF experience worse outcomes than those undergoing AVR for non-Class I indications. In fact, subclinical irreversible myocardial damage may occur in clinically well-compensated and closely monitored patients while awaiting formal surgical indications. This condition could be prevented by the use of multimodal imaging parameters, in particular longitudinal LV strain and magnetic resonance imaging. In addition, better cut-off values for mortality predictors should be established. This review aims to identify simple models that integrate several echocardiographic and cardiac magnetic resonance-derived parameters to predict the optimal timing of surgical treatment in asymptomatic patients with chronic severe AR.

## 1. Introduction

Aortic regurgitation (AR) is characterized by the diastolic reflux of blood from the aorta into the left ventricle (LV). The overall prevalence of AR detected by color Doppler echocardiography in adults was reported to be 12–15% [1,2], with most cases being trace or mild. Moderate or severe AR is less common, occurring in 1.6% of cases. A community-based study reported a 4.5% prevalence of moderate or severe AR in patients aged over 65 years [3]. The most frequent causes of primary valve disease are bicuspid aortic valve, rheumatic disease, infective endocarditis, and calcific and myxomatous degeneration, while fenestrations are less frequent [4,5,6,7]. Secondary common causes include the dilation of the ascending aorta, as seen in patients with degenerative aneurysms, aortic genetic disorders, and aortic dissection.

Transthoracic echocardiography (TTE) is the first-line diagnostic modality used to assess the mechanism, the grade of severity, the degree of secondary left ventricle (LV) remodeling, and any hemodynamic conditions associated with this valvular disease [8]. AR mechanisms follow Carpentier’s functional classification. Type I is characterized by normal cusp motion, aortic root dilation, or leaflet perforation. Type IIa is defined by leaflet prolapse with excessive motion and Type IIb by free edge fenestration in a cusp, both resulting in an eccentric jet. Type III is characterized by restrictive leaflet motion [9,10,11].

The 2020 American College of Cardiology/American Heart Association (AHA-ACC) guideline [12] for valvular heart disease (VHD) features a four-stage classification (Table 1) of AR based on the symptoms, anatomy, and hemodynamic effects of valvular disease.

AHA/ACC, European Society of Cardiology (ESC), and Japanese Society of Cardiology (JSC) guidelines for the diagnosis and management of AR contain recommendations that do not always concur [12,13,14]. It is important to note that many of the current recommendations are based on small dated studies conducted more than 20 years ago [15]. Over the last decade, more accurate imaging modalities, advances in surgical techniques, and the improved quality and durability of prosthetic valves have placed the guideline’s recommendations under the scope. As a result, the optimal timing of surgical interventions for asymptomatic severe AR with a preserved LVEF remains uncertain considering that the natural history of this condition may not be as benign as previously thought. Severe AR is associated with significant cardiovascular morbidity and mortality even in asymptomatic patients. Mortality rates can be as high as 19% within 6.6 years of diagnosis [16]; Detaint et al. reported a 10-year survival rate of 78%, suggesting an annual mortality of 2.2% compared with 0.2% reported in previous studies [17]. Therefore, clinical advances in the diagnosis and management of the disease are paramount.

The purpose of this review is to highlight new parameters and integrated models using imaging techniques to improve AR severity quantification and the detection of subclinical LV damage to predict the optimal time for surgical treatment in asymptomatic patients with chronic severe AR.

## 2. Quantifying Aortic Regurgitation: Lights and Shadows

### 2.1. Echocardiography

Transthoracic echocardiography is the preferred imaging modality for initial assessment. However, despite the availability of multiple echocardiographic parameters [18,19], most have drawbacks and show low inter-agreement results that may limit accurate AR quantification in routine clinical practice.

There are qualitative, semiquantitative, and quantitative methods using 2D-TTE and Doppler techniques.

### 2.2. Semiquantitative and Qualitative Criteria

- Vena contracta (VC): it is the narrowest jet width measured at the level of the aortic valve just below the flow convergence region. It provides an estimation of the size of the effective regurgitant orifice area (EROA) [12,20]. However, it can be affected by factors such as the presence of multiple jets, a variety of regurgitant orifice shapes (often elliptic or irregular) and leaflet calcification. When reliable, this parameter is very useful, and a value > 6 mm is associated with severe AR.
- Proximal regurgitant jet width and proximal regurgitant jet width to the left ventricular outflow tract (LVOT) diameter ratio: these are two of the first semiquantitative parameters reported. They are both measured immediately below the aortic valve. A ratio greater than 65% [21] or a jet width > 10 mm [22] is indicative of severe AR. However, limitations in the eccentric jet and the distal expansion of the regurgitant jet question the general use of this parameter.
- Continuous wave Doppler: it is an indirect assessment of AR severity, which is significantly influenced by LV compliance as well as LV and aorta pressures. The deceleration rate and pressure half-time reflect both the degree of regurgitation and ventricular end-diastolic pressure. As AR progresses, the late diastolic jet velocity decreases, and the pressure half-time shortens. A pressure half-time < 200 ms is consistent with severe AR; however, this method is more useful in acute rather than chronic AR [18,20].
- Diastolic flow reversal in the descending aorta: this is a qualitative parameter described for diagnosing severe AR using pulsed wave Doppler placed just distal to the origin of the subclavian artery. An end-diastolic velocity measured at peak R wave > 20 cm/s or a diastolic velocity–time integral ≥ 15 cm are signs of severe AR [23,24]. In young patients, this cut-off value should be increased to 17–18 cm given the elastic recoil of the normal aortic wall. In addition, holodiastolic flow reversal in the abdominal aorta is also a specific sign of severe AR. However, these methods can be influenced by the diastolic period, aortic distensibility, and dilatation [25].

### 2.3. Quantitative Criteria

The best parameter for quantifying AR would be that capable of measuring the total amount of regurgitant volume (RegVol) or regurgitant fraction (RegF), which is a percentage of the total stroke volume that returns to the ventricle during diastole.

Several quantitative echocardiographic parameters can be assessed using the continuity equation or the analysis of the proximal isovelocity surface area (PISA method) to estimate EROA and RegVol. Imaging of the flow convergence zone is obtained from all echo views, but it has several limitations due to different convergence angles and the interposition of valve tissue. In addition, obtaining a correct hemisphere of the convergence zone with the PISA method is more difficult than in mitral regurgitation [26].

Severe AR depends on EROA, mean regurgitant velocity (usually high in chronic conditions), and the duration of the diastole as RegVol decreases at higher heart rates [27]. EROA is less affected by hemodynamics and is a feasible parameter for AR grading in both central and eccentric jets. An EROA ≥ 30 mm^2^ or RegVol ≥ 60 mL indicates severe AR. A RegF (RegVol/LVOT stroke volume) > 50% is also a sign of severe AR [20], even if EROA and RegVol are in the moderate range in patients with an impaired LVEF.

The prognostic value of these thresholds, which have been shown to outperform semiquantitative methods [17,28,29], has been validated by some expert groups. However, in patients with eccentric regurgitant jets or valve calcifications, good-quality continuous and color Doppler recordings are frequently difficult to achieve [30,31]. Furthermore, the Doppler volumetric method used to calculate RegF is time-consuming and susceptible to multiple small measurement errors, which can result in substantial overall inaccuracies. The potential for error accumulation using a multi-variable equation to calculate EROA makes this method less advisable for individuals without a high level of echocardiographic experience and, thus, has significant limitations in routine clinical practice [25]. Therefore, no single measurement of Doppler parameters is sufficiently precise to quantify AR in individual patients; thus, an integration of multiple parameters is required [32,33].

Three-dimensional color Doppler echocardiography enables the visualization of the VC in simultaneous orthogonal views and allows for an assessment of its cross-sectional area [33,34], improving the accuracy of AR quantification, particularly in patients with eccentric jets [34,35]. Several studies have shown that a three-dimensional VC measurement is highly accurate, reproducible, and superior to the PISA method [34,36].

Transesophageal echocardiography is essential to improve accuracy in patients with poor echogenicity. However, the primary utility of this technique is to evaluate the AR mechanism and identify predictive markers of repairability and postoperative outcomes.

### 2.4. Cardiac Magnetic Resonance

**Figure 1:**
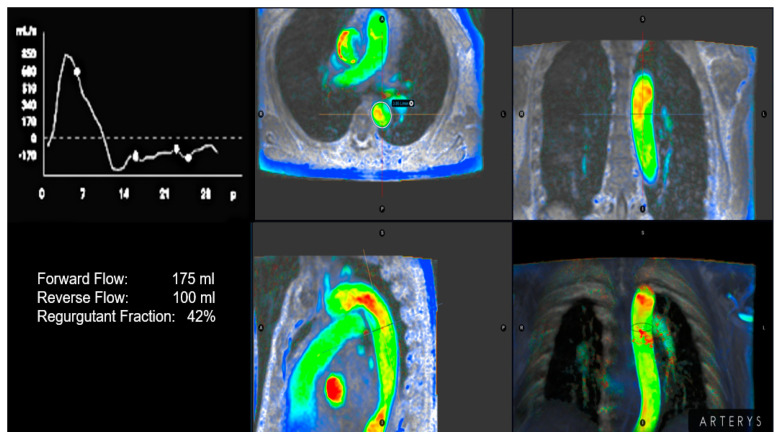
Quantification of aortic insufficienciy based on color-encoded four-dimensional plasecontrast images. The aortic insufficiency holodiastolic reversal flox is depicted in red in the distal portion of the aortic arc and proximal descending aorta. A regurgitant fraction of 42% was computed based on flow analysis.

Recently, there has been a growing focus on the use of cardiac magnetic resonance (CMR) for grading AR. The quantification of RegVol and RegF at the sinotubular junction is highly reproducible and not limited by acoustic windows [37,38,39]. In addition, the presence of holodiastolic regurgitant flow in the descending aorta is another method for AR quantification. Recently, a study [37] revisiting the echocardiography of patients with chronic AR identified 40% of cases that could be re-classified according to severity grading under the scope of CMR. This finding was particularly important, particularly for patients diagnosed with moderate-to-severe AR using echocardiography. A RegF of ≥27% best discriminated significant from non-significant AR; furthermore, holodiastolic regurgitant flow in the descending aorta and N-terminal pro-B-Type natriuretic peptide (NT-proBNP) was significantly more likely to reach the combined endpoint (heart failure, hospitalization, and cardiovascular death) in the multivariate model [37]. A RegVol > 40 mL and RegF ≥ 30% with CMR have been proposed to define severe AR, as they have a stronger correlation with echocardiography. These CMR thresholds are significantly lower than the criteria established in the echocardiographic recommendations (RegVol ≥ 60 mL and RegF ≥ 50%) and could explain the mismatch between moderate-to-severe AR using 2D echocardiography and mild-to-moderate AR using CMR [38]. The diastolic reversal velocity in the descending aorta and 3D vena contracta area (VCA) determined with echocardiography had the strongest correlation with CMR-derived RegVol and RegF in patients with chronic severe AR [30,38]. It is important to note that the best reproducibility was obtained using the sinotubular junction as the anatomical landmark. Four-dimensional flow MRI has potential advantages over phase-contrast MRI; nonetheless, further studies are needed to optimize its clinical use in AR quantification [40] (Figure 1).

**Figure 2:**
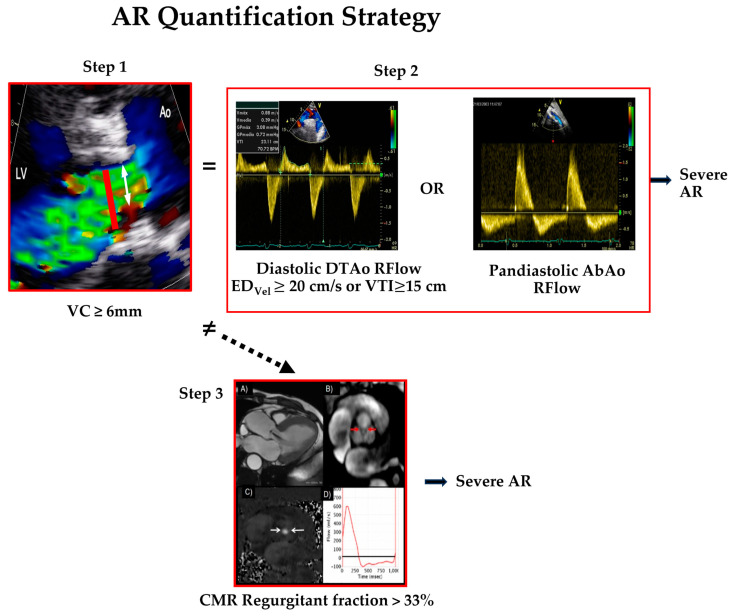
Aortic regurgitation (AR) quantification strategy: severe AR is diagnosed with Doppler echocardiography when vena contracta (VC) by colour Doppler is ≥6 mm (white arrow in Step1) and end-diastolic velocity ≥ 20 cm/s or VTI ≥ 15 cm in reversal flow of proximal descending thoracic aorta or pandiastolic reversal flow in the abdominal aorta are present (Step2). In cases with disagreement between these steps, a regurgitant fraction > 33% using CMR confirms the presence of severe AR (Step3). Abbreviatures: ED_Vel_ = end-diastolic velocity; VTI = velocity–time integral; R Flow = reversal flow; DTAo = proximal descending thoracic aorta; AbAo = abdominal aorta.

In summary, the most reliable approach for assessing the severity of AR is to incorporate different Doppler methods [13]. Grading AR severity is straightforward when the results of different parameters agree, especially jet and VC widths as well as the holodiastolic reverse flow in the proximal descending and abdominal aorta. When parameters afford conflicting information or there are discrepancies between AR severity and left ventricular parameters or symptoms, CMR could be the technique of choice to define AR severity, LV volumes, and LV function (Table 2, Figure 2).

## 3. Left Ventricle Size in Aortic Regurgitation Evolution: Victim of Parsimony?

Chronic severe AR imposes a significant combination of volume and pressure overload on the LV that, in the early stages, results in compensatory structural changes in the myocardium [41]. In this context, diastolic dysfunction precedes systolic dysfunction [42]. Preload refers to the mechanical stretch and tension experienced by the myocardium during the diastolic phase. In response to volume overload, the myocardium undergoes chamber remodeling characterized by eccentric hypertrophy. This means that the heart cavity dilates while the wall thickness decreases or remains unchanged. Patients can remain asymptomatic for a long time during this stage [43]. As the disease progresses, the balance between chamber enlargement and wall thickening may be not preserved, resulting in uncompensated wall stress and a maladaptive response. This change in heart phenotype is known as pathological remodeling. The precise timing and mechanisms of remodeling during the compensated and uncompensated stages of volume overload have not been fully established, as remodeling is a *continuum*; further investigation is required. Several molecular, metabolic, mechanical, and hemodynamic responses characterize a subclinical LV dysfunction stage. When compensatory mechanisms are overwhelmed, resulting in decreased stroke volume, systolic dysfunction develops and mortality increases significantly [44], leading to the conclusion that the LV is the victim of the silent passage of time in chronic AR. Unfortunately, in many cases, surgical intervention is indicated too late to definitively alter the patient’s prognosis; therefore, it is crucial to identify the exact moment just before irreversible changes in myocardium begin to occur [45].

## 4. Is Ejection Fraction the Only Prosecution Witness?

### 4.1. Left Ventricular Ejection Fraction (LVEF) 2D vs. 3D

This is the central parameter used to diagnose LV systolic dysfunction; it is also a good predictor of adverse outcomes in patients with severe AR. Old studies have shown that an LVEF < 50% was an important predictor of complications. However, more recent, larger studies have demonstrated that poor postoperative outcomes are expected when the LVEF falls below 55% [45,46]. Assessing a correct LVEF can be challenging due to poor image quality, the need to infer ventricular shape to calculate a 3D volume from a 2D image, and the high dependence on load variations. All these factors make the method prone to low inter- and intra-observer reproducibility, even repeated EF measurements in the same individual may result in a variability of 5–7% [47]. Three-dimensional echocardiography offers several advantages over 2D techniques. The acquisition of real-time volumetric data eliminates the need for geometrical assumptions. However, current 3D technology also has limitations, such as reduced image quality and a lower frame rate than 2D echocardiography.

CMR is the reference standard for measuring the LVEF by calculating the volume from equally spaced slices in end-diastole and end-systole, without requiring any geometrical assumptions. Significant differences in normal volume measurements between echocardiography and CMR have been reported. CMR has lower intra-observer and interobserver variabilities compared with TTE and may have a better predictive value of outcomes [48]. The reproducibility and resolution of echocardiography techniques have improved, approaching the accuracy of CMR, with the introduction of contrast-enhanced 2D and 3D echocardiography [49]. However, the two methods cannot be considered interchangeable due to the current lack of evidence.

### 4.2. Left Ventricular End-Systolic Dimension

This is a useful measure, as it incorporates both systolic function and volume overload components. It has been associated with clinical outcomes in asymptomatic patients; however, they are underestimated by echocardiography compared with CMR [50]. LV remodeling progresses gradually, and the LVEF remains well preserved in patients with stage B AR when the volume and pressure overload have not yet caused significant hemodynamic changes. For several years, LV volumes have been reported by echocardiographic laboratories; the American Society of Echocardiography and the European Society of Cardiovascular Imaging (ASE/EACVI) [12,13] jointly published criteria for the severity of LV dilation based on the LV end-systolic volume index (iLVESV); however, guideline recommendations for surgery are still based on linear measurements of the LV end-systolic diameter (LVESD) and iLVESD, as there is insufficient outcome evidence based on LV volumes. Yang et al. [51] recently reported the association between the iLVESV and survival in 492 patients with asymptomatic moderate-to-severe AR. They observed increased mortality at iLVESV values of 40 mL/m^2^ and a significant mortality risk when the iLVESV exceeded 45 mL/m^2^. Nonetheless, it is remarkable that the iLVESV did not clearly outperform the iLVESD in terms of mortality risk. Similar results were published by the same authors in a larger series showing an excellent reproducibility of LV volumes using TTE [52]. However, this reproducibility was evaluated in acquired images when the main variability was generated using TTE imaging acquisition. More evidence is required to support these results before this parameter can be generalized to indicate surgery for individual patients in routine clinical practice.

### 4.3. Myocardial Contractile Reserve

A contractile reserve under exertion evaluated using stress echocardiography has been shown to predict postoperative LV recovery and is independently associated with the deterioration of symptoms or LV systolic function in asymptomatic patients with severe AR and preserved LV systolic function [53]. Some echocardiographic parameters, such as the LV contractile reserve [54], iLVESV, and global longitudinal LV strain (LV-GLS) on exertion [55,56], have been proposed as predictors of complications. In asymptomatic patients, peak oxygen consumption is associated with a large left ventricular end-diastolic volume (LVEDV), and higher levels of NT-proBNP were independently associated with poorer exercise capacity and oxygen uptake [57]. Therefore, the role of exercise testing must be individualized. It may be helpful when there is a discrepancy between the clinical presentation and resting echocardiographic findings [58]. However, clinical decisions should not be based solely on changes observed from stress echocardiography, as these indices have not been adequately validated.

### 4.4. Global Longitudinal Strain

**Figure 3:**
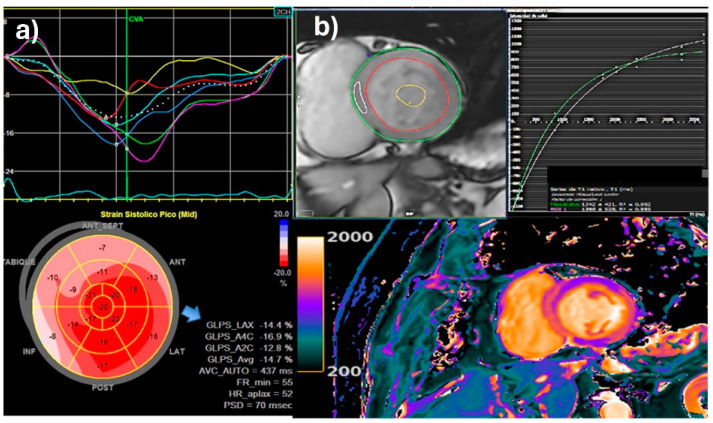
(**a**) Reduced global longitudinal strain (GLS) in patient with severe aortic regurgitation and preserved LVEF. LV-GLS = −14% (blue arrow); (**b**) native-T1 mapping value calculation: MOLLI sequence in a chronic severe aortic regurgitation. T1 mapping values are higher in the basal septum (pink ROI) compared with global myocardial values, consistent with myocardial interstitial fibrosis in the septal wall. Images processed with CMR 42^®^ Circle CVI.

Speckle tracking describes the motion of myocardial acoustic speckles in three spatial directions and provides information about regional myocardial strain as a surrogate of LV deformation. Strain imaging is suggested to be an additional tool to identify subclinical ventricular dysfunction in the setting of severe AR with a preserved LVEF. In this valvulopathy, the longitudinal orientation of myocardial fibers in the subendocardial layer renders decreased longitudinal contraction, an early sign of LV dysfunction. The LV-GLS correlates with the LVEF; however, it may be more sensitive in detecting subclinical myocardial dysfunction in the presence of a pathological process such as AR even when the EF is normal. Several observational studies have demonstrated that the LV-GLS is an independent predictor of mortality in patients with severe AR [59,60,61,62]. Lower strain values were associated with disease progression and impaired outcomes of surgically treated patients. It was also found to be an independent predictor of mortality and may play a role in determining AR surgery indication [61,63]. In a large study of 1063 asymptomatic patients with severe chronic AR with a preserved LVEF and iLVESD < 25 mm/m^2^, the LV-GLS demonstrated incremental prognostic value for longer-term survival. In a follow-up to the prior study, the LV-GLS had a prognostic value postoperatively, with impaired LV-GLS values both immediately following surgery and persistently after intervention being associated with increased long-term mortality [63]. A systematic review reported that worse LV-GLS values were associated with poor cardiovascular outcomes [64]. In a larger study [61] published by Alashi et al., the LV-GLS cut-off values that discriminated favorable and unfavorable outcomes were −19%, yet normal LV-GLS values in healthy subjects obtained with the same technology are −17.3 ± 2.5%. In a subgroup that returned for follow-up examinations, a persistently impaired LV-GLS was associated with increased mortality. Indeed, another large study carried out at the Mayo Clinic on 550 patients with asymptomatic moderate-to-severe or greater AR explored the impact of an automated LV-GLS on survival, comparing it with conventional LV parameters. They reported that an LV-GLS threshold of <15% alone or combined with an iLVESV ≥ 45 mL/m^2^ was significantly associated with increased mortality risk; thus, it could be considered in early surgery decision-making [65] (Figure 3a). The main limitation of strain is that speckle tracking acquisition and analysis algorithms differ between vendors, and there is no well-agreed cut-off value for this parameter to define systolic dysfunction. The speckle tracking technique should be used in conjunction with 3D echocardiography to overcome the inherent shortcomings of conventional 2D speckle tracking.

### 4.5. Myocardial Work

Both the LVEF and LV-GLS are influenced by loading conditions, particularly afterload. Noninvasive LV myocardial work is a novel parameter of LV myocardial performance [66,67], myocardial deformation, and noninvasive blood pressure as a surrogate of afterload. Patients with chronic moderate or severe AR and a preserved LVEF had a preserved or increased LV global work index and LV global constructive work with preserved LV global work efficiency despite the moderate impairment in the LV-GLS. Interestingly, the postoperative impairment of the LV global work index, observed in 28% of patients, was closely associated with worse LV reverse remodeling and could be related to more extensive myocardial fibrosis.

### 4.6. Myocardial Fibrosis

In recent years, there has been particular interest in the tissue characterization data afforded by CMR to identify advanced disease stages linked to adverse clinical outcomes, mainly for the detection and quantification of myocardial fibrosis [67,68]. For this purpose, the method includes late enhancement (inversion recovery) sequences to assess focal or replacement myocardial fibrosis 10–15 min after intravenous gadolinium injection, as gadolinium exhibits a longer washout period compared with viable myocardium. Also, T1 mapping techniques are incorporated to quantify extracellular matrix expansion without a contrast injection based on the patient’s hematocrit, pre-contrast or native T1 value, and post-contrast value. It represents a surrogate of diffuse fibrotic transformation, which is partially reversible in the earliest stages until it develops into scar tissue with a strong correlation with a histopathological valvular heart analysis (Figure 3b).

**Figure 4:**
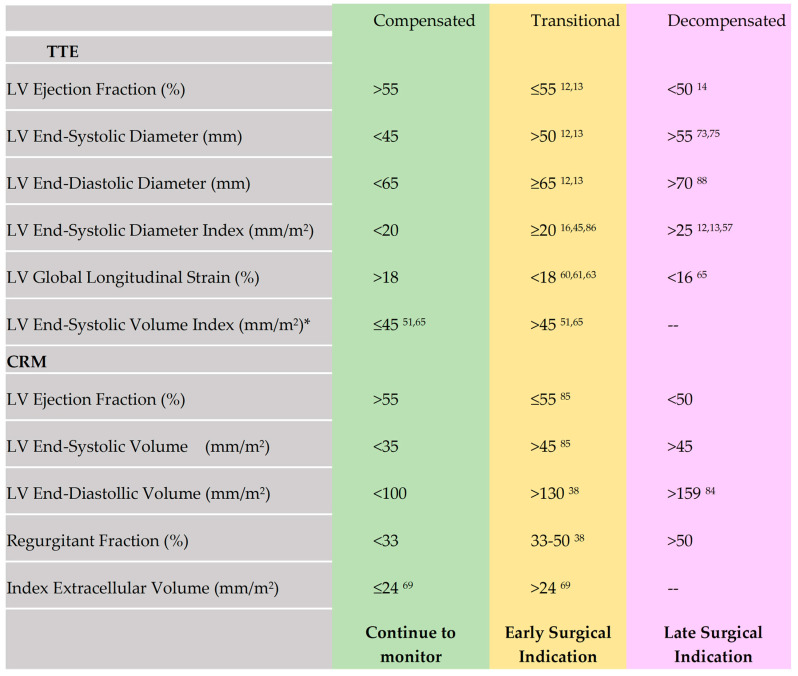
LV size and function in the three stages of chronic severe AR. Decision for surgical treatment in asymptomatic patients: continue to monitor (green), early indication of surgery (yellow), and late surgical indication (pink) based on established and emerging TTE and CMR parameters. * measurements can be precise and reproducible when performed by highly experienced centers.

LV remodeling in AR is associated with regional replacement fibrosis directly imaged using late gadolinium enhancement (LGE), as previously discussed. For diffuse interstitial fibrosis, two surrogate biomarkers of CMR have emerged: global extracellular volume (ECV), which measures the proportion of the LV extracellular matrix, and an indexed ECV (iECV), which refers to the absolute volume of the LV myocardium that is extracellular, divided by body surface area (Figure 4). The detection of preoperative LGE levels at baseline has been associated with persisting symptoms, poor LV recovery, compromised event-free survival, and an increased risk of mortality in surgical AR cohorts [69,70]. Senapati et al. [71] studied a large cohort of 177 chronic AR patients via CMR T1 mapping. They found that iECV rather than ECV was associated with both the need for valve surgery and mortality. In this study, AR RegF severity ≥ 30% and an iECV ≥ 24 mL/m^2^ presented the worst outcomes, supporting the evidence that the transition period from adaptive to maladaptive remodeling and to irreversible damage has significant implications in the timing of therapeutic interventions in chronic AR. On the other hand, in Lucas et al. [72], LGE areas were present in 31% of patients with AR, mostly non-ischemic (90%), suggesting that replacement fibrosis may not be uncommon in severe AR. The study found that the amount of preoperative diffuse fibrosis was higher in patients with AR than with aortic stenosis. After aortic surgery, patients showed iECV regression, indicating a decrease in total absolute diffuse fibrosis. Nevertheless, greater iECV values were maintained in the AR group, suggesting a sustained difference in fibrosis and greater LV structural changes compared with the aortic stenosis group. Regarding diffuse myocardial fibrosis in AR, patients had similar ECVs before and after surgery. This stability suggests balanced postoperative reductions in myocyte mass and the extracellular matrix. The greater diffuse fibrosis seen in patients with AR may reflect the different signaling pathways activated when both pressure and volume overload are present. The authors highlight that diffuse fibrosis is a plastic condition, which can regress after surgery, and the importance of CMR parameters in predicting LV reverse remodelling.

In conclusion, CMR is a useful tool in borderline asymptomatic patients in whom detecting subtle changes in LV volumes or EF as well as myocardial fibrosis can contribute to modifying surgical time decisions. Histological changes in the myocardium resulting from varying degrees of myocardial insult have an impact on conventional LV parameters, such as diameters, thickness, and the LVEF. These changes are specific, reproducible, and significant prognostic biomarkers that should be considered when making therapeutic decisions.

### 4.7. Biomarkers

Brain natriuretic peptide (BNP) has been related to echocardiographic AR severity, worse functional class, poor exercise capacity, lower maximum oxygen uptake, and greater iLV-ESV. Abnormal levels at rest may indicate subclinical LV dysfunction. The combination of echocardiographic assessments and BNP measurements increases the risk stratification power. Pizarro et al. [57] studied a prospective cohort of 294 consecutive patients with severe asymptomatic AR and an LVEF > 50% to validate a BNP cut-off value for the prediction of adverse outcomes and to assess the prognostic significance of changes in BNP levels observed between baseline and one year of follow-up. They found that a cut-off point ≥ 130 pg/mL had the power to discriminate between outcomes in a subgroup of patients at higher risk of adverse outcomes without being affected by other parameters, e.g., LV diameters or EROA. In fact, in a multivariate analysis, BNP elevation could discriminate the prognosis better than echocardiographic parameters related to volume overload, suggesting that BNP is not merely a surrogate of volume overload. Persistent BNP elevation during serial follow-up has been associated with adverse clinical outcomes after an average interval of 15 months, indicating that earlier surgical intervention may be protective for these pre-symptomatic patients.

## 5. Too Green or Too Ripe? Timing of Surgery

The goals of the operation are to improve outcomes, diminish symptoms, prevent the development of postoperative heart failure and cardiac death, and avoid aortic complications. Despite extensive research on this topic, the timing of aortic valve replacement/repair (AVR) is a continuing challenge. A long asymptomatic interval associated with adverse LV remodeling in response to insidious disease progression complicates the optimal timing of an intervention, which must be prevented by establishing better cut-off values for mortality predictors (Table 3). The functional class, EF, and end-systolic dimension are the most consistent predictors. Based on robust observational evidence, the ACC/AHA, the ESC, and the JSC guidelines [14] recommended similar indications for surgical interventions for severe AR.

Symptom onset is an indication for surgery irrespective of LV function. When LV systolic function is normal and the patient experiences symptoms, every effort should be made to clearly relate the symptoms to the AR. When the symptoms are mild, such as New York Heart Association functional (NYHA) class II dyspnea, clinical judgment is necessary. In this setting, the role of exercise testing is especially valuable. However, in patients with progressive LV dilatation or EF decline in serial studies, the onset of mild symptoms is a clear indication for valve replacement.

For symptomatic patients with reduced LV systolic function (subnormal EF), surgery is clearly indicated. Postoperative survival is likely to be worse, and the likelihood of the recovery of systolic function is lower in patients with preoperative NYHA functional class IV symptoms or with extremely enlarged ventricles (>55 mm at end-systole) and/or very poor EF (<30%) [73,74,75]. However, even in very ill patients, AVR and subsequent medical treatment are a better alternative than long-term medical therapy alone or cardiac transplantation. Surgical series have demonstrated an improvement in postoperative survival for patients with AR and severe preoperative LV dysfunction, which reinforces this opinion [76,77,78]. Multimodality imaging, including cardiac MRI, can provide important information on postoperative management [79] by evaluating the degree of preoperative cardiac damage.

In asymptomatic patients, a surgical intervention is indicated when there is LV systolic dysfunction. The AHA/ACC guideline [12] suggests a threshold of an LVEF ≤ 55% when no other cause accounts for LV systolic dysfunction, while the JCS guideline [14] uses a threshold of <50%. The ESC guidelines support either a resting LVEF of ≤50% or <55% when the surgical risk is low. When the LVEF remains normal, LV dilatation is an indication for surgery; however, the thresholds vary across guidelines. Both the AHA/ACC and ESC guidelines agree that an LVESD threshold of >50 mm or 25 mm/m^2^ may be considered in patients with low surgical risk based on the progressive decline in the LVEF or progressive LV dilatation into the severe range (left ventricular end-diastolic diameter—LVEDD > 65 mm). The ESC guideline also supports surgical intervention when the iLVESD is 20 mm/m^2^ and surgical risk is low. The JCS states that surgery is reasonable when the LVESD is >45 mm and may be considered when the LVEDD is >60 mm or the iLVESD > 25 mm/m^2^. Interestingly, Park et al. [80] provided evidence that the LVESD (≥45 mm) was a good predictor of postsurgical mortality, and a recent publication suggested that an iLVESD of 20 mm/m^2^ could be a more appropriate value [45].

The initial fibrotic transformation of apoptotic myocardial cells in the LV is partially reversible until diffuse interstitial fibrosis develops into scar tissue, as described previously. An iECV provides a more accurate picture of remodeling changes occurring in progressive AR that lead to cellular and extracellular expansion [70]. Therefore, CMR may help stratify disease risk to optimize surgical timing with the assessment of extensive fibrosis and myocardial dysfunction.

Several studies have investigated the impact of AVR on LV abnormalities [81,82]. Surgery may result in persistent LV dilatation and systolic dysfunction and could even be associated with sudden death during follow-up periods [72]. Zhang et al. [81] investigated the AVR postoperative course in patients with an LVEDD > 65 mm and the long-term prognostic impact of postoperative LV reverse remodeling. The results showed that patients with LV remodeling had better clinical outcomes compared with those without, who had more rehospitalizations, lethal ventricular arrhythmias, or heart failure. Therefore, the no-remodeling group was at high risk and would not recover after AVR. In these cases, it is important to consider information regarding the detection of large scars or fibrosis with CMR and coronary artery disease. Under these circumstances, a transcatheter aortic valve implantation (TAVI) may have a role to play in the future.

Zhao et al. [82] assessed the effects of the LVEF on moderate AR and clinical outcomes in 1211 patients. The study also evaluated the incremental value of the LVEF in risk prediction and the effectiveness of AVR in prognostic improvement among patients with moderate AR and LV systolic dysfunction. Surgery was associated with a reduced risk of death or heart failure compared with medical therapy alone in patients with an LVEF of 35–55%. The prognostic benefit was significantly attenuated when the LVEF was <35%. Previous studies reported that patients with severe AR and severe LV dysfunction (LVEF ≥ 35%) could still derive substantial prognostic improvements from surgery with an acceptable surgical risk; however, the benefits of surgery in moderate AR may not be as significant as in severe AR and may no longer outweigh the associated risks. It is essential to discuss each patient within a heart team, including the cases with moderate LV dysfunction who still have reversible interstitial fibrosis. LV size and function in the three stages of chronic severe AR based on established and emerging TTE and CMR parameters are specified in Figure 4.

## 6. The Patient, the Valve, the Left Ventricle, and the Aorta

Indications for medical treatment in chronic AR are limited. Medical therapy, especially angiotensin-converting enzyme inhibitors (ACEIs) or dihydropyridines, may provide symptomatic improvement in individuals with chronic severe AR in whom surgery is not feasible. In a clinical trial that included 95 patients with chronic severe AR and normal LV systolic function, treated with nifedipine (20 mg/12 h), enalapril (20 mg/24 h), or a placebo, vasodilator therapy did not reduce or delay the need for AVR after seven years of follow-up. Furthermore, such therapy did not reduce the aortic regurgitant volume, decrease the size of the LV, or improve LV function [83]. Therefore, its use is not recommended for this indication. However, systemic arterial hypertension with chronic AR should be treated with vasodilator therapy. Dihydropyridine calcium channel blockers or ACEIs/angiotensin receptor blockers (ARBs) are preferred.

The timing of intervention in chronic AR patients is a delicate balance between preventing irreversible myocardial damage and avoiding unnecessary surgical risk. In this equation, patient characteristics such as age, comorbidities, other cardiovascular diseases, and individual surgical risk should be considered. In addition, the size of the ascending aorta should always be assessed using echocardiography. In cases with a diagnosis of aortic dilation or where images are suboptimal for measurement, CMR or computed tomography angiography should be indicated [4]. This approach is particularly important in patients with genetic aortic diseases, bicuspid aortic valve (BAV), or suspected functional AR. Depending on the diagnosis, surgical treatment of the aortic root or the ascending aorta may be a priority despite the absence of LV repercussions [4]. CMR should be used to assess AR severity when echocardiographic parameters are equivocal or there is a discrepancy between AR severity and LV size/function or clinical symptoms.

The adverse perioperative and long-term outcomes associated with advanced stages of the disease with irreversible myocardial fibrosis highlight the importance of timely interventions. As previously mentioned, myocardial fibrosis detection with CMR could justify earlier surgical interventions, when interstitial fibrosis is reversible.

In asymptomatic patients with severe VHD in whom the LV remains compensated (stage C-1), exercise stress testing can be used to assess whether asymptomatic patients with AR can tolerate activities of daily living and to establish the baseline level of exercise capacity, although a decrease in exercise capacity can be a signal for requiring surgery [12,13]. Furthermore, evidence of subclinical myocardial dysfunction may be actively searched using myocardial strain, CMR with LGE or T1 mapping, and a BNP/NT-proBNP levels. In patients with severe AR, exercise stress testing may define equivocal symptoms and differentiate asymptomatic from symptomatic patients. Furthermore, LV volumetric data derived from echocardiography (iLVESV ≥ 45 mL/m^2^) and CMR (LVEDV > 246 mL [84] and regurgitant fraction > 33%) can be used to identify patients at increased risk of clinical progression [85,86,87,88]. Most guideline cut-off values are based on outdated studies from a time when operative mortality rates were as high as 10%. However, in recent years, there has been a reduction in both mortality and postoperative complications. Therefore, newer strategies are necessary to identify patients who could potentially benefit from accurate risk stratification and earlier intervention. Recent studies suggest that strict adherence to the guidelines [89,90,91] may result in missing the window for early intervention in a significant number of patients. As a result, many patients will continue to experience systolic dysfunction, leading to a higher adjusted mortality rate post-surgery [45,92]. Unfortunately, until now, strain and CMR parameters were not included in the AHA/ACC or ESC guidelines. A recent publication proposed [87] a combined model, including one parameter of AR assessment (CMR RegVol, RegF, or 3D VC area) with one parameter of LV remodeling (iLVEDV assessed with CMR or GLS or E-wave obtained using 2D echocardiography) and BNP, as a good predictor of complications, which was significantly better than each parameter alone. Therefore, multimodality and multiparametric models combining two imaging indices with natriuretic peptides are highly accurate in identifying early disease decompensation. Anand et al. [93] evaluated a setting of machine learning-based algorithms to predict mortality in patients undergoing an echocardiographic assessment of moderate or severe AR. Variable selection was undertaken via a 10-fold cross-validation using a random survival forest model. The main key variables identified included age, body surface area, body mass index, diastolic blood pressure, NYHA class, AVR, comorbidities, EF, end-diastolic volume, and end-systolic dimension. The concordance index for predicting the survival of the best-performing model was 0.84 and 0.86 at one and two years, respectively. This technique could also be applied to identify high-risk patients who would benefit from early intervention.

Surgery is the primary treatment for severe AR and involves AVR. However, valve repair may be considered for aortic valves with favorable morphological characteristics and good expected durability in experienced referral centers. TAVI may also be evaluated by a heart team in selected patients with contraindications for valve replacement [94].

## 7. Conclusions

The management of severe AR is influenced by several factors, including patients’ characteristics, ascending aorta dilation, and LV size and function. However, the current challenge is to improve the accuracy of AR severity quantification and to establish more sensitive markers of subclinical myocardial damage. The most reliable approach to assess AR severity is to incorporate different Doppler methods, especially jet and VC widths, as well as the diastolic flow reversal in the proximal descending and abdominal aorta. CMR is the preferred technique when there are discrepancies among Doppler echocardiographic methods. Recent studies have shown the benefits of indicating surgery earlier than recommended by the guidelines. New parameters such as the LV-GLS with echocardiography and CMR-derived indices of LV volumes and myocardial fibrosis should be incorporated into clinical practice to define the optimal timing of surgical treatment in asymptomatic patients with chronic severe AR.

## Figures and Tables

**Table 1:** Stages of Aortic Regurgitation.

Compensated Stage		Decompensated Stage	
No subclinical LV dysfuncion	Subclinical LV dysfunction	Reversible	Irreversible
Normal LVEF and stroke volume		Decline in LVEF	
Normal LV compliance filling pressure	Reduced LV-LGS Increased BNP	Reduced LV compliance Elevation of LV filling pressure Reduced LV-LGS Increased BNP	
Mild to moderate LV eccentric hypertrophy	Reversible diffuse myocardial fibrosis detection by CMR: T1 mapping ECV	Irreversible replacement fibrosis: LGE by CMR	
Asymptomatic		Symptomatic	

LV-LGS: Left Ventricle—Longitudinal Global Strain, BNP: Brain Natriuretic Peptide, ECV:Extracelular Volume, LGE: Late Galdolinium Enhancement, CMR: cardiac magnetic resonance.

**Table 2:** Grading the severity of chronic AR with echocardiography.

	**AR severity**		
	Mild	Moderate	Severe
TTE			
Semiquantitative parameters:			
VCW (cm)	<0.3	0.3–0.6	>0.6
Jet width/LVOT width, central jets (%)	<25	25–45 46–64 ^a^	≥65
Jet width (mm)	<5	5–10	>10
Reversal diastolic flow in proximal DAo, PW (VTI cm)	<10	10–15	>15
Reversal diastolic flow in AbAo, PW (VTI cm)	-	-	Holdiastolic ^+^
Jet deceleration rate, CW (PHT, msec)	Incomplete or faint	Medium, 500–200	Steep, <200
	Slow, >500		
Quantitative parameters: ^++^			
Regurgitant Volume (mL/beat)	<30	30–44 45–59 ^a^	≥60
Regurgitant Fraction (%)	<30	30–39 40–49 ^a^	≥50
EROA (cm^2^)	<0.10	0.10–0.19 0.20–0.29 ^a^	≥0.30
CMR			
Regurgitant Fraction (%)	<20	20–33	>33
Regurgitant Volume (mL)	<30	30–45	>45
Reversal diastolic flow in proximal Dao	-	-	Holodiastolic ^+^

AbAo: Abdominal aorta; CWD: continuous wave Doppler; DAo: descending thoracic aorta; EROA: effective regurgitant orifice area; LVOT: left ventricular outflow tract; PHT: pressure half-time; PW: pulsed wave Doppler; VCW: vena contracta width; VTI: velocity-time integral. ^+^ Very high specificity; ^++^ Only with high quality image and by expert groups; ^a^ Moderate AR is subclassified in mild-to-moderate and moderate-to-severe.

**Table 3:** Main subclinical myocardial dysfunction evaluation in asymptomatic patients with chronic aortic regurgitation.

Diagnostic Tests	Parameters
Speckle tracking echocardiography	LV-LGS
Stress echocardiography	Contractile reserve Unmask symptoms
Biomarkers	BNP NT-proBNP
T1 mapping CMR	Difusse reversible fibrosis Focal fibrosis

LV-LGS: Left Ventricle-Longitudinal Global Strain; BNP: Brain Natriutretic Peptide.
